# Early clinical experience with Guidezilla for transradial interventions in China

**DOI:** 10.1038/s41598-018-23633-7

**Published:** 2018-04-03

**Authors:** Min Ma, Kai-yue Diao, Xiao-jing Liu, Yong He

**Affiliations:** 1 Department of Cardiology, West China Hospital, Sichuan University, 37 GuoXue Street, Chengdu, 610041 China; 2 Department of Cardiology, the Sixth People’s Hospital of Chengdu, Chengdu, 610051 China; 3 Department of Radiology, State Key Laboratory of Biotherapy, West China Hospital, Sichuan University, Chengdu, China; 4 Laboratory of Cardiovascular Diseases, Regenerative Medicine Research Center, West China Hospital, Sichuan University, Chengdu, China

## Abstract

Anatomic variations, calcified, tortuous, angulated lesions, and lack of support to increase the complexity of transradial intervention (TRI). Guidezilla is a mother-and-child catheter enabling increased support during complex interventions. As there are few published reports of its use, we describe our experience using this device to assist TRI in Chinese patients. The aim of this study was to investigate the efficacy and safety of the Guidezilla guide extension catheter in complex coronary interventions. Thirty-two patients’ clinical characteristics, angiographic details, and in-hospital outcome retrospectively collected between June 2015 and August 2017. Patients were 59.44 ± 10.48 years of age and 26 (81%) were men. The most frequent target vessels were the RCA (34%) and LAD (31%), patients had complex type C (53%) or B (47%) lesions, severely tortuous (41%) and angulated (22%).With the use of Guidezilla, technique success was 100%, and procedural success was 94%. The mean diameter of the deployed stents was 2.97 ± 0.37 mm, and the length was 27.19 ± 8.14 mm. The estimated mean distance of Guidezilla intubation into the target vessel was 7.66 ± 2.29 cm.The Guidezilla catheter extension safely facilitated successful completion of TRI in complex coronary artery lesions. This device can help interventionalists successfully perform difficult procedures.

## Introduction

Transradial intervention (TRI) is often chosen for percutaneous coronary interventional (PCI) procedures because of fewer complications, earlier patient mobilization, and improved clinical outcomes compared with the transfemoral approach^1–5^. However, in many patients, the size of the radial artery limits the guide catheter (GC) to a size no larger than 7F. Also procedures involving complex coronary artery anatomies, extreme vessel tortuosity, calcification, angulation, and chronic total occlusions (CTO) are often time consuming and challenging^6,7^. TRI procedures can fail because of arterial spasms, failure to puncture the access site, failure to cannulate the lesion vessels, and lack of adequate guide support. Most of these obstacles can be dealt with by certain tips and tricks. One solution is use of a guide catheter extension system (GCES).

**Figure 1:**
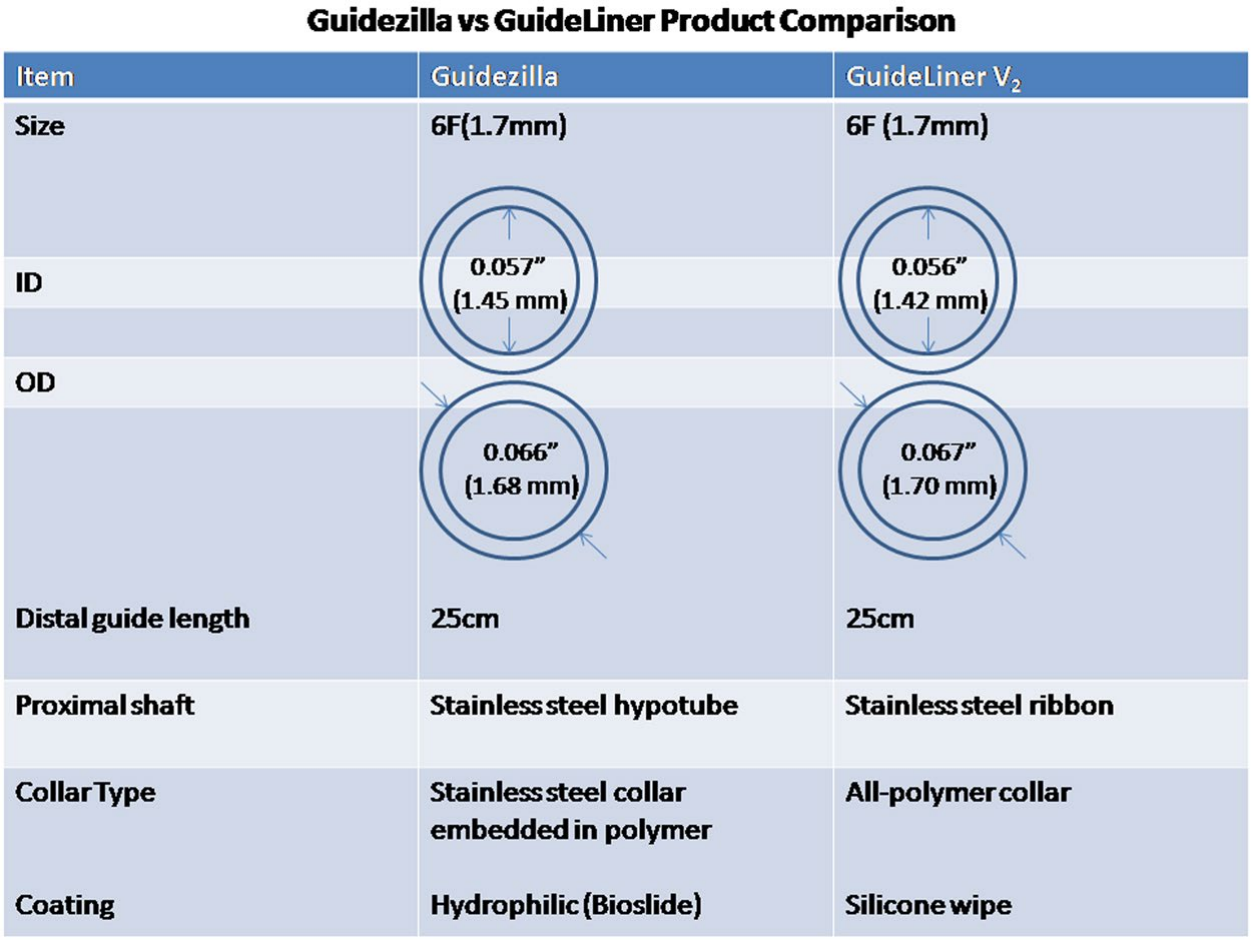
Characteristics of the Guidezilla and GuideLinerV_2_. ID = inner diameter, OD = outer diameter.

Commercially available GCES include the Proxis device (St Jude Medical, St Paul, MN, USA), Heartrail II catheter (Terumo, Tokyo Japan), GuideLiner (Vascular Solutions, Minneapolis, MN, USA), and Guidezilla (Boston Scientific, Natick, MA, USA). The Proxis device and the Heartrial II catheter are 120 cm catheters that are introduced into the mother guide and require removing the Y-connector^8–11^. The GuideLiner catheter is a rapid GCES with a long flexible tubular end that can be deeply advanced into lesions, providing support without the need to disconnect it from the mother guide^12–14^. The Guidezilla GCES is a unique, rapid exchange mother-and-child catheter. For difficult or complex procedures such as anchor balloons, deep seating, buddy wire technique, super-stiff guide wires, or GC change to a more supportive configuration^15–17^, the Guidezilla is simpler, quicker, more effective especially for transradial PCI, where the GC lacks support. Guidezilla, has a 25 cm catheter with a polymer proximal collar, and a 1.45 mm inner-diameter that has more room to deliver intervention device, and the 1.68 mm outer diameter reduces GC interference, which is smaller than the GuideLiner (Fig. 1). The braid design facilitates additional back-up support for complex or tortuous vessels, calcification, and angulated coronary anatomy lesions. The Food and Drug Administration approved Guidezilla in July 2013^18^, and the a limited number of published reports of its use for complex PCI found it to be noninferior to the other three guide extension catheters.

Thus, the purpose of this study was to describe our preliminary clinical experience with Guidezilla for back-up support and balloon and stent delivery in the treatment of complex coronary lesions via the transradial approach report at a single center in China.

## Methods

### Study population

Thirty-two consecutive patients with coronary artery disease (CAD) and TRI procedures utilize Guidezilla at between June 2015 and August 2017. All had undergone angiography and all had failed conventional techniques. The study was approved by the ethics committee of West China Hospital and complied with ethical principles of the Declaration of Helsinki. All patients gave written informed consent before enrollment.

### Baseline characteristics

Patient demographics, target vessel, ACC/AHA lesion type^19^, lesion complexity, access site, procedure success and complications, and in-hospital outcome were recorded. Data collected and reviewed by two cardiologists experienced in analysis of quantitative coronary arteriography. Indications for use of the Guidezilla GCES included: (1) anomalous or angulated take-off of native coronary arteries, (2) proximal tortuous vessels, (3) extreme calcification or long lesions, (4) for back-up support of delivery of balloons, stents, rotational atherectomy or aspiration devices, (5) or failure with other techniques. Success was defined as stent placement in the target lesion with residual stenosis of less than 20% and TIMI 3 flow. Procedural success was defined as technique success without major Guidezilla-related complications. Deep intubation was defined as depth more than 2 cm.

### Interventional procedures

Transradial PCI techniques with Guidezilla were performed by experienced interventionalists following standard clinical protocols with right radial artery access and a 6F GC and radial sheath (Terumo, Japan). Dual antiplatelet drugs were administered orally before performing PCI. Intra-arterial nitroglycerin (200 µg) and unfractionated heparin (70–100U/kg) were administered during the PCI procedure after achieving arterial access. The choice of other drugs, intervention approach, equipment, and technique was also at the operator’s discretion. Selective coronary artery angiography (CAG) was performed using diagnostic 5F multifunctional catheters (Terumo, Japan). The 6F radial sheath was removed immediately after the PCI procedure, and a radial compression bandage was used to prevent bleeding of the access puncture site. The bandage was removed after 6 h, provided no bleeding occurred.

### The Guidezilla catheter and its use

We used the 6F version Guidezilla GCES that has two radiopaque marker bands to increase visibility and facilitate accurate placement and positioning in the aorto-ostium of the coronary arteries. The radiopaque safety tip is soft and flexible, which reduces the risk of vessel damage and complications. A hydrophilic surface coating provides a smooth finish that reduces friction. The procedures began by positioning the GC and advancing the guide wire (GW) across the target lesion. The Guidezilla was then advanced over the GW through the hemostatic valve of the Y-connector to intubate the target lesion. When the Guidezilla was in position, balloons, and stents were delivered over the same initial GW. This technique permitted deep intubation with back-up support and great coaxial alignment. In patients where a kissing balloon was needed, a wire exchange was performed followed by balloon dilation of the side branch through the stent struts. Before final inflation of the kissing balloon could be performed, the Guidezilla had to be removed.

### Statistical analysis

This was not a comparative study. Continuous variables were expressed as means and standard deviation (SD) and categorical variables were expressed as number and percentage (%). Data analysis was performed with SPSS software version 19.0 (IBM Corp., Armonk, NY, USA). All authors take responsibility for all aspects of the reliability, interpretation, and freedom from bias of the data.

## Results

### Study population and baseline characteristics

A total of 32 consecutive procedures were performed using Guidezilla. Baseline clinical characteristics of the patients are presented in Table 1. They were 59.44 ± 10.48 years of age, 26 (81%) were men, and the majority had risk factors, including hypertension (75%), hypercholesterolemia (34%), current smoking (59%), and diabetes mellitus (47%). About 31%, 47%, 28% had previous coronary revascularization, CAD and myocardial infarction (MI), respectively. Fifty-nine percent of the patients presented with unstable angina pectoris.

**Table 1:** Patient demographics and clinical characteristics.

Variable	N(%) or Mean ± SD
Age(years)	59.44 ± 10.48
Male/Femal	81/19%(26/6)
**Risk factors**	
Hypertension	75%(24/32)
Hypercholesterolemia	34%(11/32)
Current somking	59%(19/32)
Diabetes mellitus	47%(15/32)
Prior PCI	31%(10/32)
Prior CAD	47%(15/32)
Prior MI	28%(9/32)
**Indication for PCI**	
ST-elevation MI	16%(5/32)
Non-ST-elevation MI	13%(4/32)
Unstable angina	59%(19/32)
Stable angina	13%(4/32)

Abbreviations: PCI = percutaneous coronary intervention, CAD = coronary artery

disease, MI = myocardial infarction.

### Interventional procedures and indication for Guidezilla

Guidezilla GCES only through TRI access was used in 30 of the 32 procedures (94%). Both through the TRI and femoral access was used in 2 procedures (6%), because the 2 cases with chronic total occlusion (CTO), which need both transradial and transfemoral approaches to achieve contralateral injection or retrograde manipulation.

**Figure 2:**
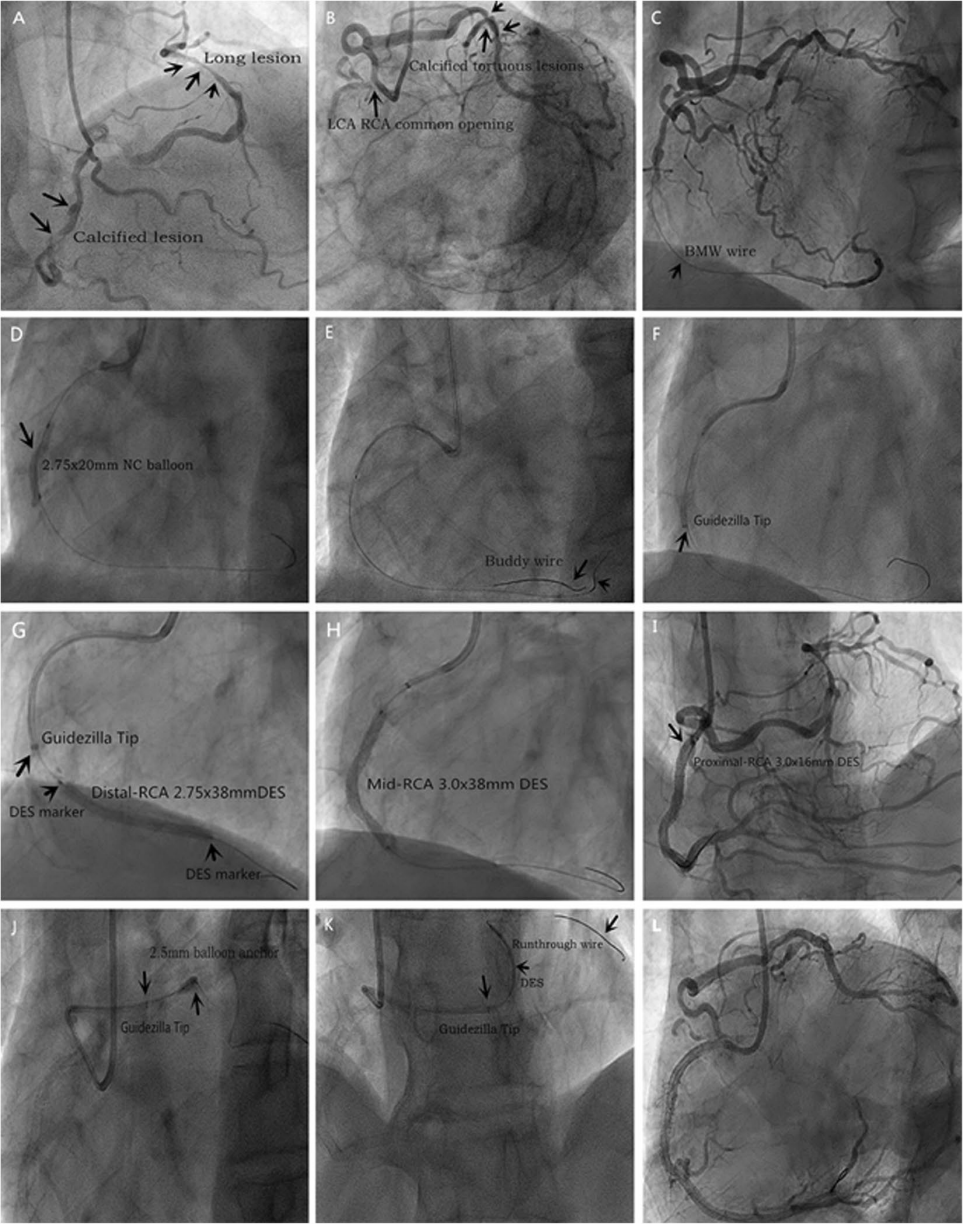
Successful treatment of coronary lesions with Guidezilla in elder patient with CKD. (**A**,**B**) showing outside medical coronary angiogram results. (**C**) BMW wire to the distal vessel of RCA. (**D**) Stenosis of lesions were predilated with 2.75 mm × 20 mm NC balloon. (**E**) Use the“buddy”wire to improve GC coaxial and support. (**F**) Guidezilla introduced into the RCA successfully. (**G**–**I**) Promus Premier DES were deployed from the distal to the proximal RCA. (**J**) 2.5 mm anchor balloon technique. 6F Guidezilla was introduced into the distal LCX without difficulty. (**K**) 2.75 mm × 32 mm Promus Premier DES was delivered and was post-dilated with a 3.0 mm × 12 mm NC balloon. (**L**) The final coronary angiography shows a good result.

The target vessel was the RCA (34%) and LAD (31%). Patients had complex type C (53%) or B (47%) lesions, severely tortuous (41%) and angulated (22%). Details of the procedures are shown in Tables 2 and 3. The major finding was that severe tortuosity (41%) and angulation (22%) predicted the need for Guidezilla support, suggesting that vessel characteristics could be the primary consideration for use of active guide support in TRI procedures. Technique success was achieved with this GCES technique in all 32 procedures (100%); procedural success was achieved in 30 of 32 (94%). In one procedure, proximal dissection of the RCA occurred with high-pressure balloon dilation, which was not related to the Guidezilla. Stent stripping from the target lesions occurred in two patients. The mean diameter of the stents deployed was 2.97 ± 0.37 mm, the mean length was 27.19 ± 8.14 mm. The estimated mean distance of Guidezilla intubation into the target vessel was 7.66 ± 2.29 cm, and the intubation distance was ≥10 cm in eleven patients (34%). Representative images are taken during a procedure in a patient where initially it was not possible to deliver any equipment, but did succeed with the use of a Guidezilla catheter. In one elder patient CAG showed a left and right coronary common opening, left and right coronary arteries with severely tortuous, long stenotic lesions, calcification, and angulation successful treatment of coronary lesions with Guidezilla (Fig. 2).

**Table 2:** Summary of Cases performed Using Guidezilla catheter.

Case	Access	Lesion vessel	Lesion type	GC	Clinical indication	Intubation depth(cm)	Indication for use Guidezilla	Stent (mm)	Complication
1	R	LAD	Severe tortuous, B	6F EBU	STEMI	6	Balloon and stent delivery	3.0 × 24 Promus	None
2	R	RCA	Severe tortuous, C	6F JR	UA	4	Balloon and stent delivery	2.75 × 28 Promus	None
3	R	LAD	Severe tortuous/angulation, B	6F XB	Non-STEMI	7	Stent delivery	2.75 × 16 Promus	None
4	R	LCX	Distal long lesion, B	6F XB	STEMI	10	Back-up support and stent delivery	2.75 × 28 Xience	None
5	R/F	LAD LCX	Extreme tortuous/angulation, C	6FXB	UA	8	Back-up support and stent delivery	2.75 × 20 3.5 × 16 Promus	None
6	R	RCA LAD	Severe tortuous and calcified, C	6F JR	UA	6	Back-up support and stent delivery	3.5 × 20 2.75 × 16 Promus	None
7	R	RCA	Very tortuous and calcified, C	6F JR	UA	10	Back-up support	3.0 × 38 Xience	None
8	R	LAD	Mid severe tortuous, B	6F XB	STEMI	8	Balloon and stent delivery	3.0 × 18 Firdbird	None
9	R	LCX	Extreme tortuous/angulation, B	6FXB	Non-STEMI	6	Stent delivery	3.5 × 16 Promus	None
10	R	RCA	Severe calcification, B	6F JR	SAP	8	Balloon and stent delivery	2.5 × 38 Xience	Proximal dissection
11	R	RCA	Tortuous,B	6F JR	Non-STEMI	10	Balloon and stent delivery	3.0 × 18 Firdbird	None
12	R	RCA	Tortuous, C	6F XB	UA	6	Stent delivery	2.75 × 20 Promus	None
13	R	LAD	Calcificationt, tortuous, C	6F XB	SAP	12	Back-up support and stent delivery	2.75 × 28 Xience	None
14	R	LCX	Tortuous, angulation, C	6FXB	UA	10	Back-up support and stent delivery	3.5 × 16 Promus	None
15	R	RCA	Angulation, C	6F JR	UA	4	Stent delivery	2.75 × 28 Promus	None
16	R	RCA	tortuous, B	6F XB	Non-STEMI	12	Back-up support	2.75 × 28 Xience	None
17	R	LAD	Severe tortuous, B	6F XB	STEMI	8	Stent delivery	3.5 × 33 Xience	None
18	R/F	LCX LAD	Long lesion, C	6F XB	UA	10	Stent delivery	3.0 × 38 3.0 × 28 Promus	Stent stripping
19	R	RCA LAD	Calcification, distal lesion, C	6F XB	UA	6	Balloon and stent delivery	2.75 × 28 3.0 × 16 Promus	None
20	R	RCA	Extreme tortuous/angulation, B	6F JR	STEMI	8	Balloon and stent delivery	2.75 × 28 Promus	Stent stripping
21	R	LAD RCA	Severe tortuous, B	6F XB	SAP	6	Back-up support	2.75 × 29 3.0 × 15 GuReater	None
22	R	LM LAD	Severe Calcificationt, tortuous, C	6F XB	UA	8	Back-up support and stent delivery	2.5 × 38 3.0 × 38 4.0 × 28 Promus	None
23	R	RCA	Calcificationt, tortuous, C	6F JR	UA	10	Back-up support and stent delivery	3.0 × 38 3.5 × 35 3.5 × 32 Promus	None
24	R	LAD	Long lesion, C	6FJL	UA	10	Back-up support and stent delivery	2.75 × 38 2.75 × 12 Promus	None
25	R	LAD	Calcificationt, tortuous, B	6F XB	UA	7	Back-up support	2.25 × 28 3.0 × 24 Promus	None
26	R	LAD	Severe tortuous, B	6F EBU	UA	8	Stent delivery	2.75 × 29 3.0 × 15 GuReater	None
27	R	LAD	Long lesion, C	6F JL	SAP	10	Stent delivery	2.5 × 38 3.0 × 32 Promus	None
28	R	LCX	Severe calcification, B	6F XB	UA	8	Balloon and stent delivery	2.5 × 24 GuReater	None
29	R	RCA	Tortuous,B	6F JR	UA	10	Balloon and stent delivery	3.0 × 36 3.0 × 24 GuReater	None
30	R	RCA	Tortuous, C	6F JR	UA	6	Back-up support and stent delivery	4.0 × 36 GuReater	None
31	R	LAD	Calcificationt, tortuous, C	6F EBU	UA	4	Back-up support and stent delivery	2.5 × 32 3.0 × 32 Promus	None
32	R	LCX	Tortuous, angulation, C	6F XB	UA	5	Back-up support and stent delivery	3.0 × 38 Promus	None

Abbreviations: R = radial, F = femoral, R/F = switch from radial to femoral, GC = guide catheter, RCA = right coronary artery, LAD = Left anterior descending artery, LCX = left circumflex artery, SAP = stable angina pectoris, UA = unstable angina, STEMI = ST-elevation myocardial infarction, Lesion type = ACC/AHA classification.

**Table 3:** Summary of target vessels, lesions, stents, access site, depth of Guidezilla intubation and complication characteristics.

Variable	N(%) or Mean ± SD
**Target vessels**	
LAD	10/32(31%)
LCX	5/32(16%)
RCA	11/32(34%)
Multi-vessel	6/32(19%)
**Target lesions**	
Type B/C	15/17(47%/53%)
Severe calcification	5/32(16%)
Severe tortuous	13/32(41%)
Angulation	7/32(22%)
**stents**	
Mean length of stents implanted(mm)	27.19 ± 8.14
Mean diameter of stents(mm)	2.97 ± 0.37
**Access site**	
Only R	29/32(91%)
Both R and F	3/32(9%)
**Depth of Guidezilla intubation**(cm)	7.66 ± 2.29
**Complications**	3/32(15%)

Abbreviations: RCA = right coronary artery, LAD = Left anterior descending artery, LCX = left circumflex artery, R = radial, F = femoral.

## Discussion

To the best of our knowledge, this is the first report of the successful use of the Guidezilla GCES for highly complex procedures in a series of 32 patients in China. The results show that the Guidezilla was a useful and safe adjunct tool during challenging TRI procedures. Previous reports described similar techniques in smaller patient series^20,21^. Using a Guidezilla GCES within a 6F GC, we performed interventions easily without the possibility of losing access to the coronary arteries while changing out the GC. TRI with complex coronary anatomy is often challenging and time consuming. Thus, proper PCI strategy with proper coronary intervention devices is crucial to ensure the success of the procedure. The PCI procedural success rate with the use of GCES ranges from 93% to 98%^22,23^. Farooq *et al*. demonstrated that GCES may allow deeper intubation of the GC, referred to as “rail-roading”^14^. Takahashi *et al*. noted that use of GCES provides substantial improvement in back-up support for complex coronary interventions^24^. The GuideLiner GCES has been used for selective contrast injection, providing better visualization of target lesions with smaller amounts of contrast, which is described in detail in the Twente GuideLiner registry^22^. Currently, four GCES devices are commercially available, the Proxis, Heartrail II, GuideLiner, and Guidezilla. The Guidezilla became available in China only recently. In our series, the main indications for the Guidezilla GCES were: (1) anomalous or angulated take-off of native coronaries; (2) proximal tortuous vessels; (3) extreme calcification or long lesions; (4) as back-up support for delivery of balloons, stents, and other equipment such as rotational atherectomy or aspiration devices; or (5) failure using other techniques. In our example case, we could not initially to deliver any equipment, and the stent could not pass through the RCA stenosis because of tortuosity, calcification, and angulation. A 6F Guidezilla was successfully introduced into the RCA, and a 2.5 mm anchor balloon was introduced into the distal LCX without difficulty using the mother-and-child maneuver with the Guidezilla catheter to achieve a successful result. We recommend limiting the extension of the Guidezilla catheter to <15 cm beyond the tip of the GC, in order to avoid complication, operator should pay attention not to extend the whole segment in the target vessel. Guidezilla catheter segment length is 25 mm, the manufacturer’s instructions suggested extension of the catheter out of the guide catheter less than 15 cm.Otherwise, it is possible that the mother catheter and child catheter lose the coaxiality and impede withdrawal of the device. In present study, the longest intubation depth of Guidezilla into the target vessel was 12 cm as further distal advancement of the Guidezilla device could cause the entire guide segment to track outside of the guide catheter and impede withdrawal of the device, and it is important to maintain coaxial alignment. For patients with renal insufficiency and the expectation of selective angiography (e.g., in a dominant LCA system), a Guidezilla GCES may be considered as a first choice. The patient described in the present study had a baseline creatinine of 204 µmol/L with compromised renal function. PCI was completed using less than 100cc of contrast, which minimizing contrast load and decreased the possibility of contrast-associated nephropathy. The patient was discharged 3 days later and remained asymptomatic at 1-year follow up. This is in line with a report by Tunuguntla *et al*.^25^ on the use of a GuideLiner catheter to minimize contrast during PCI in a patient with chronic kidney disease (CKD).Reports of complications associated with the Guidezilla GCES are limited. We experienced coronary stent stripping in two procedures (6%) and proximal dissection of the RCA in one (3%). Stent stripping from its delivery balloon occurred on the introduction into the proximal Guidezilla catheter edge. The same event has been reported by Waggoner *et al*.^21^, who described the proximal junction transition as the site of stent stripping in their case. The stainless steel collar of the Guidezilla is not as flexible as the polymer collar of the GuideLiner, so passing through the acute angle of the aortic arch caused the stent to encounter the proximal edge of the stainless steel collar during transition to the GCES. This appears to work against introducing stents into the device when it is within an acute angulation of a vessel. In our experience the solution was to remove the Guidezilla before pulling back an unimplanted stent. Farooq *et al*.^14^, reported that there was a small risk of damage to large or bulky stents as they enter the collar. They advised using low-profile stents with the GCES, avoiding ones than 4 mm in diameter.

One of our patients experienced coronary dissection because of high pressure balloon dilation, which was not related to the Guidezilla use. Similar complications reported during GCES procedures include stent deformation or dislodgement on withdrawal, coronary artery ischemia or dissection, pressure damping, and balloon kinking^26–31^. In difficult lesions, compared with other techniques, such as anchoring balloons, deep seating, buddy wires, super-stiffer wires, or GC change to a more supportive configuration^15–17^, the Guidezilla is simple, quick, and effective. It is especially helpful in transradial PCI where GC lack passive support, or in complex procedures.

## Limitations

Our data was retrospective and obtained at a study single center, and the study sample was small, and accordingly native bias might have affected the accuracy and efficacy of our statistical analysis. However, this trial implemented rigorous inclusion criteria to reduce the risk of confounding bias. So, larger studies will be needed to further investigate our findings, meanwhile, we are expanding the sample size and performing a follow-up of these patients. Design modification of future GCES devices, especially the steel collar, may minimize the risk of stent stripping that we observed. Prospective randomized trials of the efficacy and safety of GCES techniques are warranted.

## Conclusion

The Guidezilla GCES safely facilitated successful completion of TRI in complex coronary artery lesions. Guidezilla use was associated with a highly success rate with no major complications. We recommend this device to assist in difficult lesions, particular in TRI procedures. It was also suitable for a patient with renal insufficiency. We believe this technique is a first-line choice of radial interventionalists treating patients with complex coronary lesions or CKD, reducing contrast use and total fluoroscopy time.
